# Super Sub-Nyquist Single-Pixel Imaging by Total Variation Ascending Ordering of the Hadamard Basis

**DOI:** 10.1038/s41598-020-66371-5

**Published:** 2020-06-09

**Authors:** Xiao Yu, Rayko Ivanov Stantchev, Fan Yang, Emma Pickwell-MacPherson

**Affiliations:** 1 State Key Laboratory of Power Transmission Equipment & System Security and New Technology, School of Electrical Engineering, Chongqing University, Chongqing, 400044 China; 2 Chinese University of Hong Kong, Electronic Engineering, Hong Kong SAR, China; 3 University of Warwick, Department of Physics, Warwick, CV47AL UK

**Keywords:** Imaging and sensing, Displays

## Abstract

Single pixel imaging (SPI) captures images without array detectors or raster scanning. When combined with compressive sensing techniques it enables novel solutions for high-speed optical imaging and spectroscopy. However, when it comes to the real-time capture and analysis of a fast event, the challenge is the inherent trade-off between frame rate and image resolution. Due to the lack of sufficient sparsity and the intrinsic iterative process, conventional compressed sensing techniques have limited improvement in capturing natural scenes and displaying the images in real time. In this work, we demonstrate a novel alternative compressive imaging approach employing an efficient and easy-implementation sampling scheme based on reordering the deterministic Hadamard basis through their total variation. By this means, the number of measurements and acquisition are reduced significantly without needing complex minimization algorithms. We can recover a 128 × 128 image with a sampling ratio of 5% at the signal peak signal-to-noise ratio (PSNR) of 23.8 dB, achieving super sub-Nyquist sampling SPI. Compared to other widely used sampling e.g. standard Hadamard protocols and Gaussian matrix methods, this approach results in a significant improvement both in the compression ratio and image reconstruction quality, enabling SPI for high frame rate imaging or video applications.

## Introduction

Single pixel imaging (SPI) is an emerging technique that captures scenes using a single point detector and then reconstructs the images through computational imaging approaches. It has solved many challenges where conventional multipixel imaging is technologically unavailable and provided novel ideas in fast optical imaging^1^. Prospective applications include 3D imaging^2,3^, ghost imaging^4^, infrared imaging^5–7^, fluorescence^8,9^, hyperspectral imaging^10,11^ and terahertz imaging^12,13^. The architecture of commonly used SPI systems consists of two main components: a single-pixel detector and spatial light modulator (SLM). The SLM generates and displays encoded masks of light on the object (also called structural illumination) and the single-pixel detector measures the inner product of an image and the masks sequentially^14^. The projected sampling masks used for modulating the image are encoded according to sampling matrices such as random matrices and Hadamard matrices^15^. Then the reconstruction problem is just calculating the inverse of the measurement matrix. However, to obtain a high signal-to-noise (SNR) reconstruction of an *N* pixel image, *N* measurements must be carried out. Consequently, long data-acquisition times are needed which make the SPI unable to capture rapid phenomenon and real-time imaging.

Fortunately, compressed sensing (CS) theory can provide a novel method to shorten the acquisition time by reducing the number of measurements by exploiting the sparsity of natural images^16^. However, the reconstruction problem then becomes a NP-hard combinatorial problem solved by varieties of optimization algorithms such as the widely used solver *l*_1_-magic^17^ and TVAL3^18^. Based on these methods, sub-Nyquist sampling can be achieved but the sampling ratio must satisfy the restricted isometry property (RIP)^19,20^ and is limited by the sparsity of the image:

1 $$SR=\frac{M}{N}=O\left(\alpha \,\log \left(\frac{1}{\alpha }\right)\right)$$

where *SR* is the sampling ratio, *M* is the number of measurements required for a good reconstruction, *N* is the total pixels of the image, *α* is the sparsity ratio of the image in the chosen domain. In practice, the sampling ratio is limited and influenced by noise, beam calibration and diffraction^21^. It is often not possible to obtain good images in practical SPI systems such as terahertz imaging systems when the sampling ratio is below 30%^13,22^. Also, without prior information of the scene the sparsity ratio of the image remains unknown. Hence, there is tradeoff between reconstructing high SNR images and having a low sampling ratio.

Recently, some SPI methods based on CS have been proposed to seek better performance by reducing computational time considerably without sacrificing SNR of reconstructed images whilst keeping a low sampling ratio. For sub-Nyquist sampling protocols, sampling the parts with the most amount of energy is the key to achieving good reconstruction, for example sampling only the Fourier coefficients with the largest amplitude. For that purpose, a lot of sampling techniques based on Gaussian matrix methods, discrete cosine transforms (DCT)^23^, Fourier transforms^24,25^, Morlet wavelets^26^, Hadamard matrix methods^27^ and binary matrices through deep learning^28^ have been proposed. From these, the Hadamard matrix sampling stands out because it has fast image reconstruction, its binary masks are easy to implement and it has high robustness to noise. Furthermore, it has been found that using a differential measurement of the Hadamard matrix enhances the SNR because it subtracts background noise^29^. Additionally, a significance-based ordering of the Hadamard basis provides a better reconstruction from fewer measurements^30^. Hence, different ordering of Hadamard matrices have been proposed. A “Russian Dolls” (RD) ordering Hadamard approach at 6% sampling ratio can produce similar or better image quality^30^. A “cake cutting” (CC) and “Origami pattern” (OP)^31,32^ sorted Hadamard approach can acquire high quality images of large pixel scale 1024 × 1024 with super sub-Nyquist sampling ratio even below 0.2%. Likewise, the CC and OP order and order performs better than the RD order. However, the RD order showed a saw tooth descent in the retrieved image quality and is sensitive to the environmental noise. The CC order approach needs to count the block numbers of each reshaped mask, while the RD order and OP order approach need to manipulate the elements of the Hadamard matrix, which is complex and time-consuming for large pixel resolution images, and there is no specific mathematical or physical explanation for CC. Furthermore, there is no fast construction method for RD order and OP order.

In this work, we present an efficient and easily-implemented sampling protocol for super sub-Nyquist SPI while avoiding basic presumptions that general scenes are sparse. Our approach is based on an optimized ordering of the Hadamard basis through total variation (TV) of each reshaped mask for general scenes. It also reveals the inner mathematical mechanism of this and similar ordering of Hadamard matrices. Following this idea, the most significant patterns, those with the lowest TV value, are always projected first. Reordering the Hadamard matrix proved to be more computationally efficient than the “Russian doll” and “Cake cutting” approaches. We numerically compare the reconstructed images obtained from four different Hadamard matrices and a Gaussian matrix through three image quality assessment indices. We find that, for natural images, the total variation ascending order produces better reconstruction quality than the other 4 approaches.

### Image reconstruction and assessment

The SPI with CS technique works by taking sequential measurements and can be described mathematically by (2),

2 $${{\boldsymbol{y}}}_{M\times 1}={H}_{M\times N}{{\boldsymbol{x}}}_{N\times 1}+{{\boldsymbol{e}}}_{M\times 1}$$

where ${{\boldsymbol{x}}}_{N\times 1}$ is a 1D vector reshaped from a *n* × *n* pixel image; ${H}_{M\times N}$ represents the M sampling masks each formed by reshaping one row of the measurement matrix *H*(*M* × *N*), m < N, *N* = *n×n*, ${{\boldsymbol{y}}}_{M\times 1}$ is the measurement vector of length *M* and ${{\boldsymbol{e}}}_{M\times 1}$ is the noise vector which is the same size as ${{\boldsymbol{y}}}_{M\times 1}$. Equation 2 is an underdetermined linear system of equations and the physically relevant solution is obtained by optimization methods. Here, we use the TVAL3 solver to reconstruct the image^18^. The quality of reconstruction, for thorough comparison, is evaluated by 3 indices: percentage root mean squared error (RMSE), Peak Signal to Noise Ratio (PSNR) and Structural Similarity (SSIM). All images are normalized to unity.

### Hadamard basis orders

**Figure 1:**
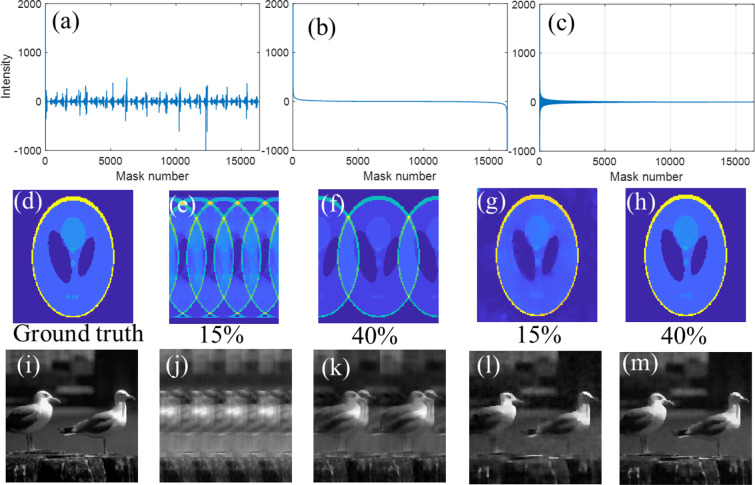
Image reconstructions with different subsets of normal Hadamard masks. (**a**) The measured signal intensity distribution of the image using normal order Hadamard. (**b,c**) are the measured signal intensity distribution in real value (*y*) descending order and absolute value (|*y*|) descending order. (**d,i**) are the ground truth of the phantom image and natural image. (**e,f,j,k**) give the reconstructed images at sampling ratio of 15% and 40% based on normal order Hadamard. (**g,h,l–m**) give the reconstructed images of the sparse image and natural image at sampling ratios of 15% and 40% based on absolute value descending order Hadamard.

A sensible assumption is that larger measured intensities contribute more to the reconstruction, therefore the corresponding masks should be retained and projected sequentially^30,31^. However, which masks carry a lot of information depends on the imaging scene and for fast changing scenes and dynamic real-time imaging this becomes even more unfeasible. There exist many different orderings of the Hadamard matrix, such as sequence order and dyadic order. However, none of them arrange the most significant patterns to appear first automatically for arbitrary scenes. In this work, we make use of the Hadamard matrix generated using the built-in function in MATLAB 2018b^33^ (otherwise known as Sylvester’s construction), which we call the ‘normal order’ to form other reordered Hadamard matrices. To test the sensing performance and reconstruction of normal order, a 128 × 128 phantom image was taken (read from the MATLAB 2018b), due to its high sparsity, and a natural scene^34^ as an example and carry out the recovery. Figure 1 shows the sensing and image reconstructions with different subsets of Hadamard masks. Our calculations were performed in MATLAB 2018b using the TVAL3 package^18^. For the sparse phantom image and natural image, we are unable to reconstruct images from this normal order at low sampling ratio. There are several overlaps in the reconstructed images, and the lower the sampling ratio is, the more overlaps and artifacts there are. Figure 1(b,c) show the measured signal intensities sorted in descending order according to their real values and absolute values. According to the two orders, we sort the normal order Hadamard rows with the new sampling masks and do the reconstructions again. Figure 1(g,h) demonstrate excellent reconstruction of the phantom image at sampling ratios of 15% and 40% with the PSNR of 30.6 dB and 47.87 dB respectively. Nevertheless, the descending order Hadamard masks do not work for the natural image (Fig. 1(i–k)) while the absolute value descending order Hadamard masks work. Figure 1(l,m) demonstrate an excellent reconstruction of the natural image using absolute value descending order at sampling ratio of 15% and 40% with the PSNR of 29.06 dB and 34.29 dB respectively. This proves the order of Hadamard masks is significant to the measurement and reconstructions, and the most significant masks should be used to sample the image first. However, in practice, it is hard to know *a priori* which mask can generate the most significant intensity values; one must perform a complete sampling and then pick up the crucial masks needed according to the measured signal.

Our goal is to produce a new order Hadamard basis based on the normal-order Hadamard matrix so that any truncation of these mask sequences will provide an optimal reconstruction. However, knowing which masks are most significant without knowledge of the imaging scene is a near impossible task. Fortunately, we can draw inspiration from the Fourier spectra of natural images: namely that low frequencies have larger amplitudes than high frequencies. Therefore, the masks with low frequencies should be measured first. Hadamard masks do not have a frequency associated with them however we can measure which masks have more sign changes. The proposed strategy is called total variation ascending Hadamard basis (TV order), and its formation rule is very simple as explained in the following. We define the sum of total variation of each row of a Hadamard matrix as:

3 $$T{V}_{i}=\sum \sqrt{{({h}_{i}{D}_{x})}^{2}+{({h}_{i}{D}_{y})}^{2}}$$

where *D*_*x*_ and *D*_*y*_ is the discretized gradient operators, which are *N* × *N* sparse diagonal matrices, for the variation in *x* direction and *y* direction respectively; *h*_*i*_ is the *i-th* row of normal Hamdard matrix; TV is the sum of variation. Afterwards, we can get the new complete Hadamard basis by sorting the normal order Hadamard basis in the ascending order of the total variation. The most important patterns come out first by using our method without prior knowledge of the object and also without complicated sorting algorithms. During the sorting process, we do not need to manipulate the elements or count the number of connected regions (also called blocks, white or black) of each reshaped row of the *N* × *N* Hadamard matrix. Here, we sort the total variation in ascending order because we hypothesize that the smaller the TV is, the more probable this mask contributes more information for the reconstruction as it can be thought of having a lower frequency. Consequently, it only takes a little more (negligible) time to reorder the normal Hadamard matrix by generating a diagonal sparse matrix and implementing matrix multiplication.

**Figure 2:**
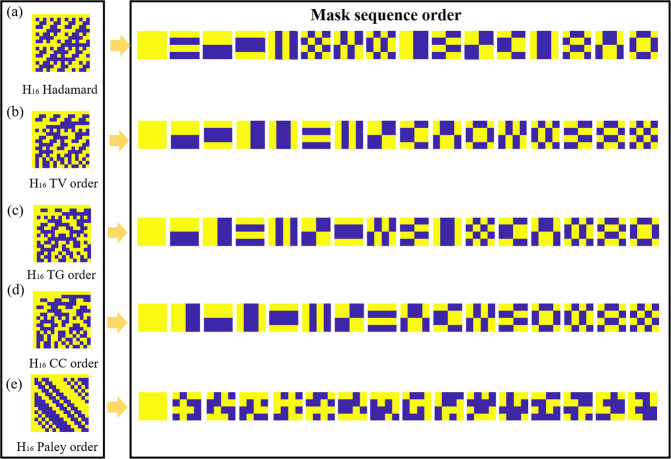
Different Hadamard ordering example. (**a–e**) are the normal order, TV order, TG order, CC order and Paley order of 16 × 16 Hadamard matrices and their mask sequence orders respectively, each mask is one row of Hadamard matrix which are reshaped into a 4 × 4 2D matrix.

**Figure 3:**
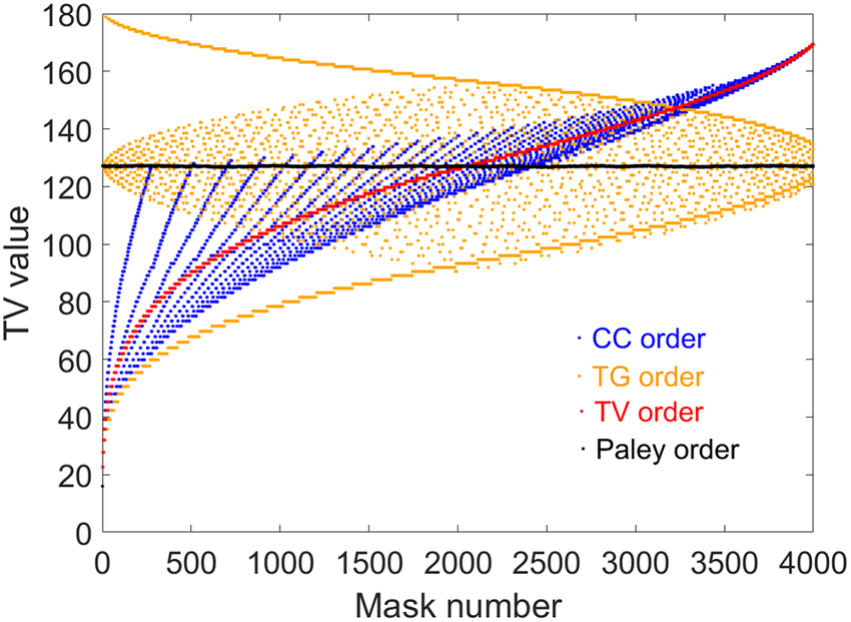
Total variation of the proposed four reordered Hadamard matrices comparison. The blue, orange, red and black dots are the total variation value distribution of CC order, TG order, TV order and Paley Hadamard matrices.

To study the inherent relation and regularity we compare 5 different Hadamard matrices: Normal order, TV order, total gradient ascending order (TG order), “Cake-cutting” order (CC order) and Paley ordering with Fig. 2(a–e) showing the different 16 × 16 Hadamard matrices that have been sorted by these methods respectively (see Methods for construction rules). Moreover, the Paley type-I Hadamard matrix is proposed to join the comparison because it has been used in single pixel imaging^27^ due to its flexibility in image size. The Paley type I Hadamard matrix is created by cyclic permutation, which in turn creates masks that are vertically, or horizontally, shifted from one another. Whilst this will not create masks that have large and small ‘frequencies’, it can still be used in under sampling by simply shifting the masks by two or three pixels each time instead of just shifting them by one pixel each time. This is what we call the Paley order. By reshaping each row of these matrices into 4 × 4 2D arrays, a complete set of the 16 masks to be projected is obtained. According to Fig. 2(b,d), the TV ordered set of 4 × 4 masks is very similar with the CC order Hadamard matrix^31^. Figure 3 shows the total variation of a 4096 × 4096 reordered Hadamard matrix (H_64_) in the four orders. The total variation of CC order and TV order has a rising trend between 16 and 180, but the CC order has some fluctuations during the rising process. The total variation of Paley order oscillates in a small range and the TG order exhibits a tendency to swing violently.

## Results

**Figure 4:**
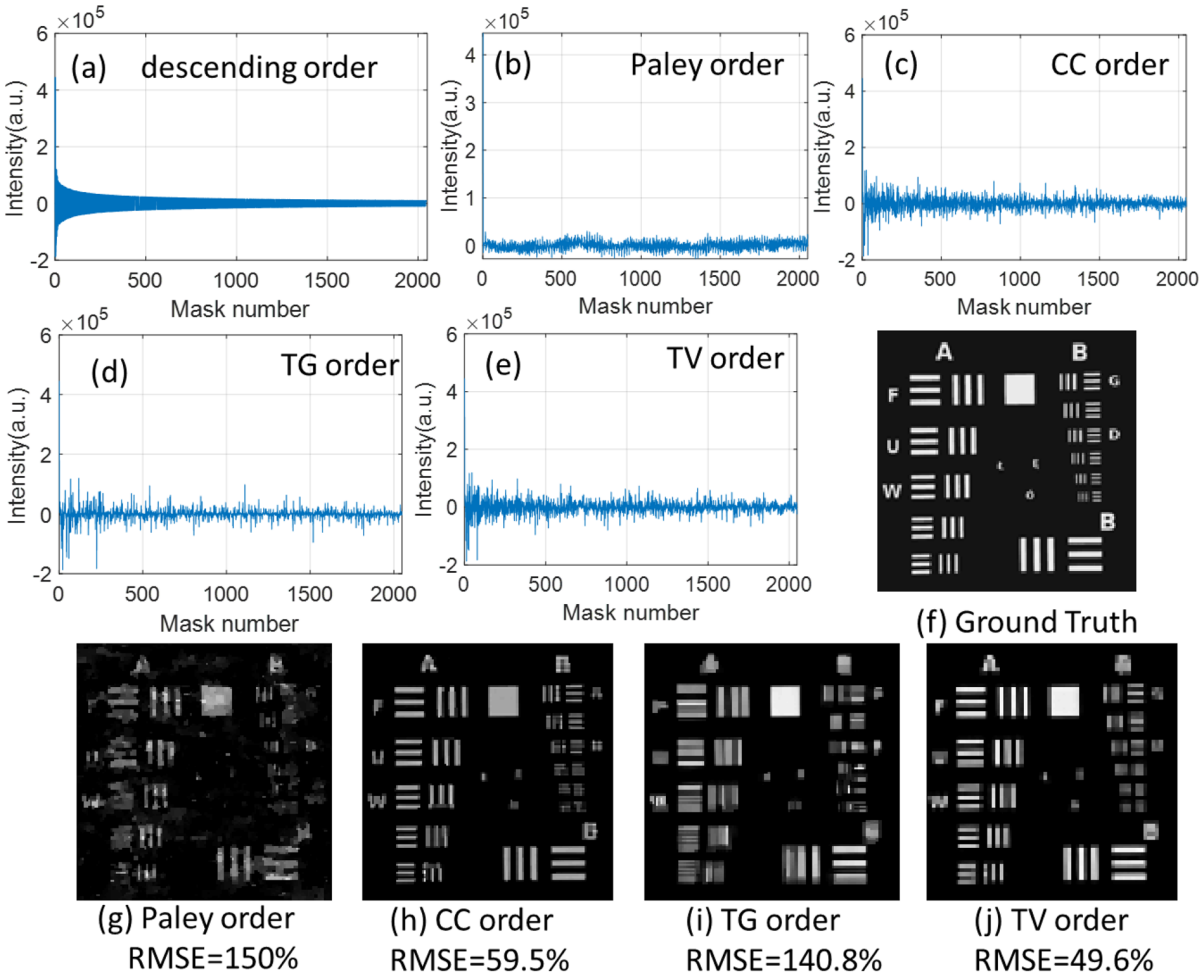
Image reconstruction with different fractions of four Hadamard basis. (**a**) is the 12.5% of full measured signal intensity sorted in descending order according to its absolute value. (**b–e**) are the measured signals intensity using subset of natural order, Paley order, CC order, TG order and TV order Hadamard basis respectively. (**f**) is the ground truth image. (**g–j**) are the reconstructed images using subset of Paley order, CC order, TG order and TV order Hadamard basis respectively.

In order to test the four approaches for image reconstruction, simulations are carried out both on sparse and natural images. One original 512 × 512 sparse image is resized to the resolution of 128 × 128 pixels as the ground truth. In this image, all values of the white letters and geometries are 0.3, while all values of the black background are 0. The sampling ratio for sub-Nyquist sampling is set to be 12.5%, hence the dimension of Hadamard matrix for this measurement is 2048 × 16384. Figure 4 gives the results of measured signal intensities and reconstructed images.

With the normal order approach, the measured signal intensities (Fig. 4(a)) distributed from 1–2048 are the most significant of intensity values. The sampled signal intensities of the Paley order approach are concentrated on the zero zone and oscillate periodically because this Hadamard is constructed from a cyclical permutation. Compared with the descending order and other four approaches, larger intensities can be seen through the denseness of the curves (Fig. 4(c–e)), especially for the CC order and TV order approaches. For the reconstruction performance, the RMSE (in percent) of the resulting images is calculated using Eqs. (5–6). Figure 4(g–j) show the RMSE of the reconstructed images at a sampling ratio of 12.5%. CC order and TV order approaches perform much better than the Paley and TG orders with the RMSE of 59.5% and 49.6% respectively. Hence, for spatially sparse images, TV order and CC order approaches both can provide excellent results.

**Figure 5:**
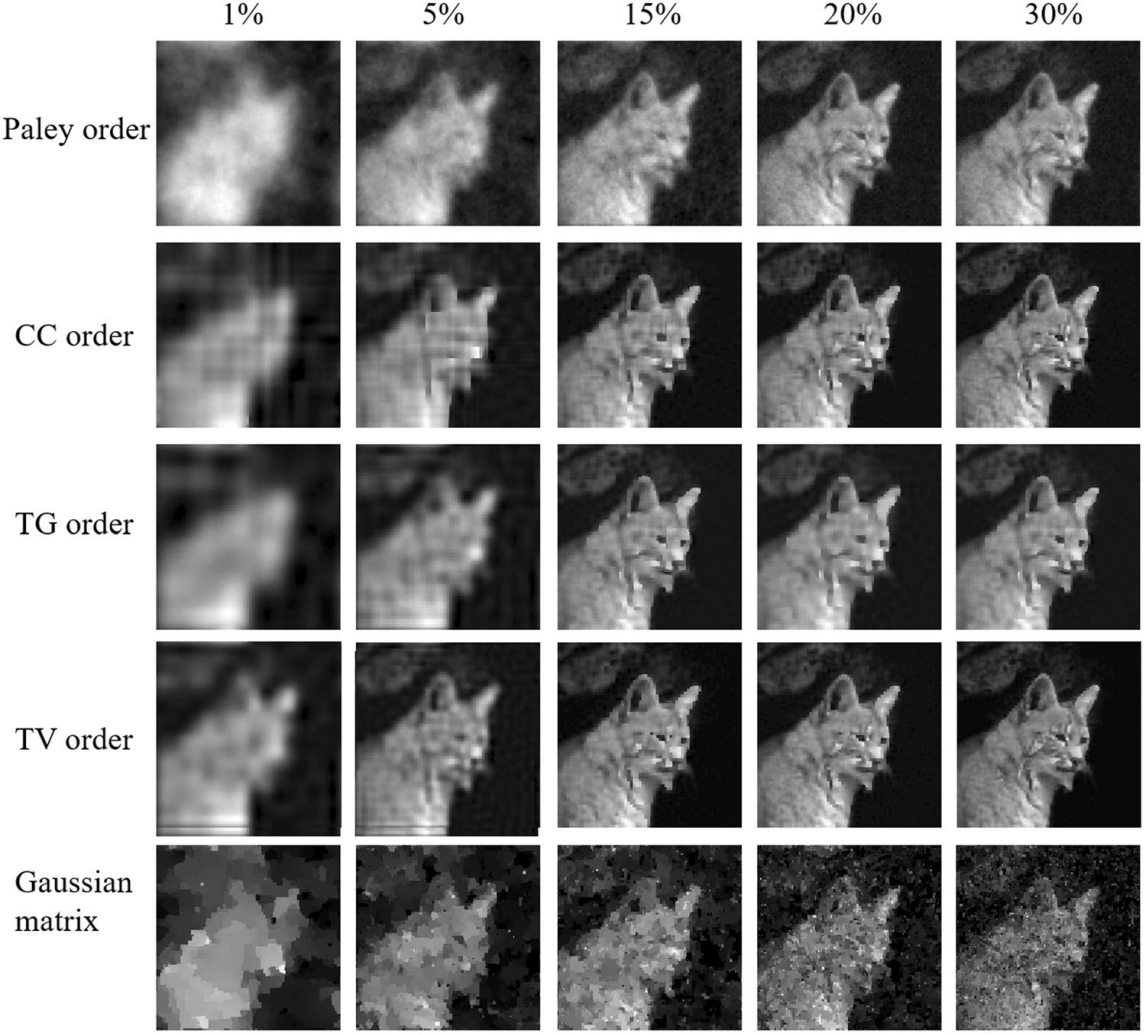
Natural scene reconstruction comparison for five approaches. From left to right, the images are reconstructed at sampling ratio of 1%, 5%, 15%, 20%, 30%.

However, imaging spatially sparse objects is not always possible or interesting, therefore we need to test the TV order approach for natural images. Due to the aim of sub-Nyquist sampling, we focus on sampling ratios between 1% and 30%. Figure 5 shows the reconstructions at different ratios. As expected, all four approaches show a similar trend with the reconstruction quality increasing with sampling ratio. The TV order approach gives significantly more details than other approaches even at the low sampling ratio of 5%. A Gaussian sampling matrix was added as it is widely used in normal compressed sensing for comparison. At a low sampling ratio, all the Hadamard matrices perform better than the Gaussian matrix.

**Figure 6:**
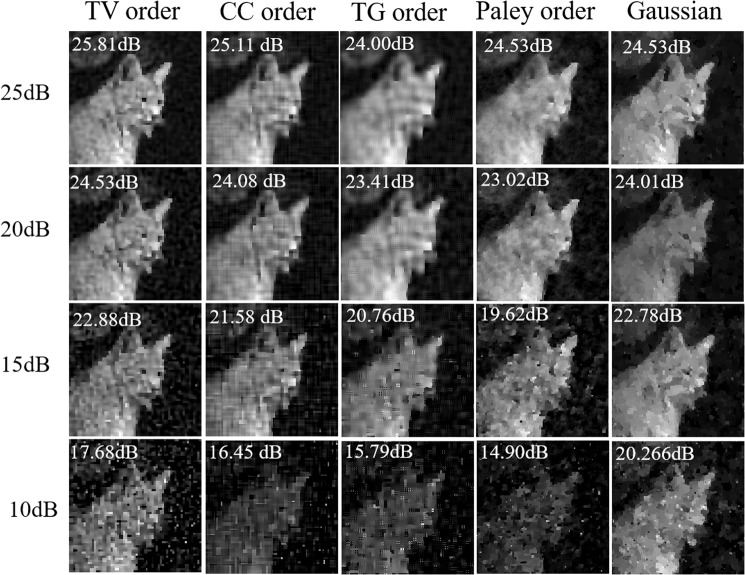
Natural scene reconstruction comparison for 5 approaches with different noise level. From left to right, the images are reconstructed at sampling ratio of 10% at the noise level of 20 dB, 15 dB, 10 dB, 5 dB.

Noise is unavoidable in real imaging systems especially when the signals are weak. We further compare the four techniques in terms of robustness to noise to emulate a real imaging system. We add white Gaussian noise to change the SNRs of the measured signal. This operation is implemented by using the *built-in* function *awgn* of *MATLAB* which the noise is the unit of *dB* using the measured signal as a reference. In other words, the smaller the noise level number is, the more noise is in the measured signal. The comparison is shown in Fig. 6 where we use a 10% sampling ratio. According to the results, all four approaches are robust to noise. Here, TV order performs better than the other three.

**Figure 7:**
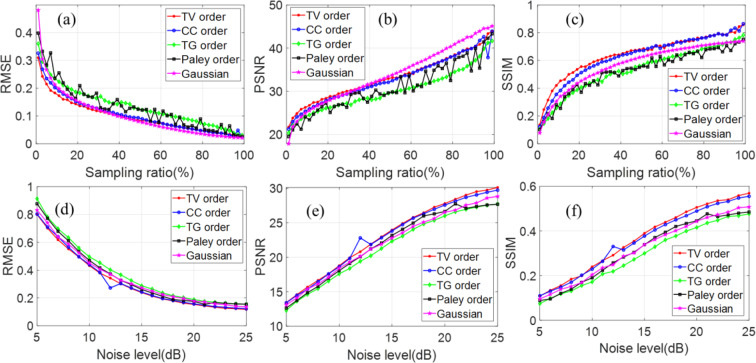
Comparison of recovered image quality of the 5 approaches without noise (**a–c**) and with noise (**d–f**).

To quantitatively evaluate the quality of a reconstructed image, we calculate the RMSE, PSNR and SSIM as a function of sampling ratio from 1%~100% at the sampling step of 2%, which is shown in Fig. 7(a–c). The three indices are computed with the ground truth as a reference. According to the results, it is clearly seen that our TV order and CC order are much better than the Paley and TG orders with an overwhelming advantage for super sub-Nyquist sampling ratio, and the TV order is better than the CC order. It should be noted that the SSIM is smaller than 1 at the sampling ratio of 100% because some information of the image is lost during the fast Hadamard transform in the TVAL3 package. As for the robustness to noise, the reconstructed images show the same falling characteristic along with increasing noise (Fig. 7(d–f)). It should be noted that the smaller the noise level (in *dB*), the more noise are added to the measured results. Nonetheless, the TV order is still better than other orders even the Gaussian matrix. Consequently, we can form an optimized reconstruction with any lower resolution using the TV ordered Hadamard basis.

As illustrated in Fig. 7(a) the RMSE of TV order across all sampling ratios is lower than the CC order which has hitherto been reported to be the best Hadamard matrix sorting method^31^.

## Discussion

**Figure 8:**
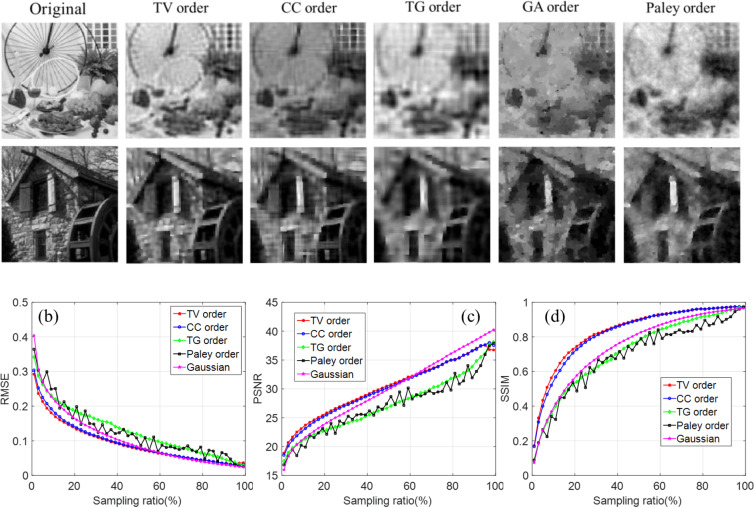
Reconstruction comparison of the 30 images. (**a**) Examples of the reconstructed images using TV order, CC order and Gaussian matrix at the sampling ratio of 10%. (**b–d**) Is the comparison of the quality of reconstructed images as a function of sampling ratio.

To verify the universality of the proposed method, we used 30 images from the dataset of standard 512 × 512 grayscale test natural images^34^ to test our method. In this simulation, the image set containing 30 images aims to simulate imaging of natural images which have a lot of variation in pixels. Figure 8(a–d) illustrates the comparison results and some examples for a 10% sampling ratio. Natural images degrade the reconstruction quality, but TV order still out-performs other methods, especially at a low sampling ratio.

**Figure 9:**
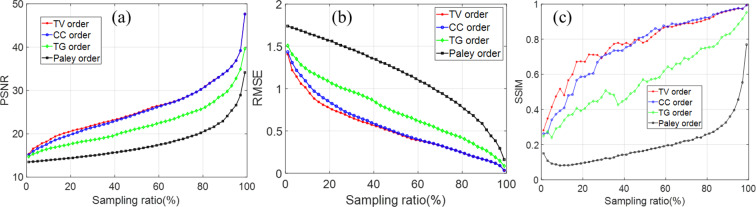
Reconstruction comparison by *l*_1_-magic algorithm as a function of sampling ratio. (**a–c**) is the RMSE, PSNR and SSIM respectively.

We used the TVAL3 algorithm which is a computationally efficient reconstruction algorithm with high reconstruction quality. It is widely used in practical single pixel imaging systems, for example it was used in the “Russian doll” order paper30, the “Cake-cutting” order paper31 and the “origami pattern” paper32. For a fair comparison and to verify the universality of our method, we also used the l1-magic algorithm to reconstruct the images using the TV order approach. Figure 9 shows the PSNR, RMSE and SSIM reconstructed by the l1-magic of the compared 4 measurement matrixes: TV order, CC order, TG order and Paley order. It is clear that the TV order outperforms the other Hadamard matrices at low sampling ratios. For the SSIM, TV order is better than CC order because the TV variation can keep more edge information.

To further analyze the inherent mechanism of reordering the Hadamard matrix, we compare the core ideas of the RD order, CC order and TV order, we find that the key point is the connected regions in the Hadamard basis pattern. From a mathematical and physical viewpoint, the total variation is the sum of the vertical variation and horizontal variation, since the Hadamard matrix only consists of 1 s and −1 s. If adjacent elements have different values, it will contribute to the increase of the total variation and the number of connected regions. From Fig. 3, the CC order method, which is an empirical method, has the most similar rising trend with the TV order apart from some saw tooth oscillations.

In a practical SPI system, the DMD projects the Hadamard basis patterns on the spatial light modulation (SLM) sequentially. For one specific pattern, the areas which have the same value (1 s or 0 s) are called connected regions in mathematical topology and will reflect the same modulation light, resulting in a coherence area. Hence, the pattern which has the smallest total variation, has the least number of connected regions as well as the biggest coherent area. Consequently, this pattern will rank in front of the ensemble of Hadamard basis patterns. For a specific object, the signal to noise ratio (SNR) of a reconstruction is correlated with the coherence light area. The relation is described as follows^35^:

4 $$SNR\propto \frac{m\cdot {A}_{coh}}{{A}_{beam}}$$

where the *m* is measurement times, ${A}_{beam}$ is the area of illumination beam, ${A}_{coh}$ is the coherence area. Hence, the bigger the total coherence area is, the more it will contribute to the measured signals and reconstruction. This mathematically explains the inherent nature of reordering the Hadamard matrix. The proposed feasibility of our method is that it can be deterministically described in mathematics and easy to calculate and implement in a practical single pixel imaging system.

Finally, Table 1 is a table of merit to highlight the improved performance by our TV order method compared to the other four approaches. The merits of all the metrics (RMSE, PSNR and SSIM) are given without noise and with 15 dB noise for sampling ratios under 20%. In this table, 1–5 are the marks for the performance of each matrix, and 5(*****) means the best. As for the Paley order and the Gaussian matrix, it is hard to say which is better because oscillations in the Paley order with increasing sampling ratio are observed while Gaussian matrix should be binarized in the practical SPI system. Hence, we ranked them only according to the reconstruction results. Generally, TV order ranks best in total and for each aspect evaluated. It should be noted that for RMSE, SSIM and implementation, the TV order is marginally better than the CC order, but for PSNR and noise robustness, the TV order approach is significantly better than the CC order.

**Table 1:** Table of Merit to compare the five approaches.

	No noise			With noise (15 dB)			Total ranking
	RMSE	PSNR	SSIM	RMSE	PSNR	SSIM	
TV order	5	5	5	5	5	5	*****
CC order	4	4	4	4	4	4	****
TG order	2	2	2	1	1	1	*
Paley order	2	2	2	3	3	3	**
Gaussian	3	3	3	2	2	3	***

### Summary

In this work, we proposed an optimized order of the Hadamard basis based on total variation ascending order for super sub-Nyquist sampling SPI. This approach utilizes a normal Hadamard basis to form an optimal Hadamard basis for any super low sampling, and it can be deterministically described by mathematics. Numerical simulations demonstrate that the TV ordered Hadamard basis outperforms the CC order and other optimized Hadamard basis matrices as well as the Gaussian matrix in image reconstruction at super low sampling both for sparse images and natural images with regards to RMSE, PSNR and SSIM. Therefore, for relatively low-resolution applications, this method is easy to experimentally implement and applicable for achieving real-time SPI.

## Methods

### TVAL3 reconstruction

TVAL3 is the acronym of Total Variation minimization by Augmented Lagrangian and Alternating Direction Algorithms^18^. The algorithm effectively combines an alternating direction technique with a nonmonotone line search to minimize the augmented Lagrangian function at each iteration. It is widely used for image reconstructions in SPI with compressed sensing techniques. We used these different Hadamard matrices as the sampling matrix. For each reordered Hadamard matrix, we take the first *M* rows as the sub-Nyquist sampling matrix, where the *M* = *N* × *N* × *SR*, SR is the sampling ratio in percentage. Each row of the sampling matrix multiplies the image which is reshaped into 1D vector to form one measured signal intensity. After all the *M* measurements, reconstructions are carried out in *MATLAB* 2018b on a laptop with 16 GB memory and 2.8 GHz CPU.

### Paley order

Paley order is formed by Paley-I type construction which is a method for constructing Hadamard matrices using finite fields^13^.

### “Cake cutting” order Hadamard basis

This strategy is to generate an optimized sort of the Hadamard basis^31^. Firstly, each row of the Hadamard matrix *H* is reshaped into a mask of *N* = *n* × *n* pixels; secondly, count the number of connected regions; finally, sort the Hadamard matrix in ascending order of their number of connected regions. In this paper, we also call the sorted Hadamard matrix the CC order.

### Total gradient ascending order

In TV order, the total variation is the variation of each row of Hadamard matrix. To further investigate the regularity of variation, we calculate the gradient of each 2D mask (*n* × *n*) reshaped from one row of a normal Hadamard matrix (*N* × *N*, *N* = *n* × *n*) and then sum all the gradient in *x* and *y* directions.

5 $$TG=\mathop{\sum }\limits_{i=1}^{N}\sqrt{{{G}_{x}}^{2}+{{G}_{y}}^{2}}$$

The built-in function *gradient* in MATLAB is used to calculate the gradient of each 2D mask. The results from the gradient function are *Gx* and *Gy* which both are 2D *n* × *n* matrix. Finally, the normal Hamdard matrix is reordered in the ascending order total gradient which is called the TG order. Different from the TV order, TG order is based on the gradient of each mask while the TV order is based on the variation of each row of the *H*(*N* × *N*) matrix.

### RMSE

RMSE is the root-mean-square error between the contaminated reconstruction image and the original image. Assuming *I* is the original image and *K* is the reconstructed image, the RMSE is defined as follow:

6 $$MSE=\frac{1}{N\times N}\mathop{\sum }\limits_{i=1}^{N}\mathop{\sum }\limits_{j=1}^{N}{|I(i,j)-K(i,j)|}^{2}$$

7 $$RMSE=\frac{MSE}{\frac{1}{N\times N}\mathop{\sum }\limits_{i}^{N}\mathop{\sum }\limits_{j}^{N}I(i,j)}$$

where *N* is the width of the images in number of pixels. *I (i, j)* is the value of the pixel in *i*_th_ row and *j*_th_ column in the original distribution, and *K (i, j)* is the value of the corresponding pixel in the reconstructed image.

### PSNR

PSNR is a full-reference image quality assessment criterion. It is the ratio between the maximum signal power and the noise power. The greater the PSNR value is, the less distorted the reconstruction images will be. In a 2-D imaging process, the PSNR is defined as follow:

8 $$PSNR=10\times {\log }_{10}\left(\frac{Max(I)}{MSE}\right)$$

### SSIM

Structure similarity index (SSIM) is calculated through mean value, square deviation and covariance of original image and recovered image. Here, we used the *built-in* function *ssim* in *MATLAB*.

## Data Availability

The datasets generated during and analyzed during the current study are available from the corresponding author on reasonable request.
